# The Effects of Foliar Sprays with Different Silicon Compounds

**DOI:** 10.3390/plants7020045

**Published:** 2018-06-07

**Authors:** Henk-Maarten Laane

**Affiliations:** ReXil Agro BV, Demmersweg 92a, 7556 BN Hengelo (OV), The Netherlands; hm.laane@rexil-agro.com

**Keywords:** foliar spray, silicates, stabilized silicic acid, silica nanoparticles, growth, yield, quality, biotic stress, abiotic stress

## Abstract

The use of foliar sprays with silicon compounds is relatively new. Initially (in 1990) foliar sprays with silicates were used. In 2003, foliar sprays with (stabilized) silicic acid were introduced, and more recently foliar sprays with silica nanoparticles have also been applied. Foliar sprays with silicates are effective as pesticides, while (stabilized) silicic acid sprays increase growth and yield and decrease biotic and abiotic stresses. The limited data on foliar silica-nano sprays show a tendency to decrease biotic stress and to stimulate a limited increase in growth and yield.

## 1. Introduction

Foliar fertilization (or foliar feeding) is the application of nutrients, plant hormones, biostimulants, other beneficial substances and pesticides to the leaves and stems of plants. The application of these substances during growth and development can improve the nutrient balance of crops, which, in turn, leads to increased yield and quality, greater resistance to diseases and insect pests and improved drought tolerance.

The literature on the application of foliar sprays starts in the 1850s [1,2] and covers 100 years or so [3,4]. From the 1950s onwards, foliar feeding in agriculture was used on an ever-increasing scale, with macro- and micro-nutrients such as nitrogen, potassium, phosphorus, zinc, copper, boron, manganese, cobalt, chromium, calcium, fluoride, iodine, iron, molybdenum, and selenium [5,6], as well as pesticides. 

During the last three decades, other substances have been used, including plant growth regulators such as abscisic acid (ABA), gibberellic acid (GA3) and biostimulants, which are applied as a foliar spray. 

Plant biostimulants are substance(s) or micro-organisms applied to plants with the aim of enhancing nutrient uptake, nutrient efficiency, tolerance to abiotic stress, and/or crop quality traits, regardless of the nutrient content [7]. These biostimulants include (a) humic/fulvic acids [8,9]; (b) protein hydrolysates and other N-containing compounds such as amino acids [8,9,10]; (c) plant and seaweed extracts [10]; (d) chitosan and other biopolymers [11]; (e) inorganic compounds including silicon [12]; and f) beneficial micro-organisms like fungi and bacteria [13]. 

By recent definition, silicon compounds are recognized and classified as biostimulants in EU regulations. Elsewhere, Si compounds are used as soil amendments and sometimes classified as fertilizers. 

Since 1990, Si compounds have been applied as foliar sprays. In this review, the effects of foliar sprays with different Si compounds will be presented. The rationale for this review is to provide an up-to-date systematic overview of the different types of foliar feeding with Si compounds [14,15]. Moreover, due to the increasing recognition of the importance of silicon products, as a result of the expanding knowledge concerning their beneficial effects (see Section 3), a review of their foliar applications is indispensable.

Due to the complexity of silicon chemistry and confusion about the different names, a survey is given of the different silicon compounds in Section 2, including a glossary. In Section 3, the various roles of silicon in plants are summarized. In Section 4 an overview of the effects of the foliar applications of different silicon compounds is provided, followed by a discussion in Section 5. 

## 2. Silicon Chemistry and Silicon in Soils

The silicon content of soils is (very) high, with an average of 28% Si by weight. The vast majority of Si compounds in the soil consists of silicon dioxide, silicate minerals and aluminosilicates, none of which are available for plant uptake. The only plant bioavailable silicon compound is monosilicic acid (MSA; synonym: orthosilicic acid: OSA), the concentration of which is (very) low in the soil. 

### 2.1. Glossary of the Various Silicon Compounds

**BAS**: bioactive silicon or biosilicon: monosilicic acid. 

**Biogenic silicon**: silicon compounds in the plant.

**Liquid silicon**: all soluble silicic acid forms, from monosilicic up to subcolloidal silicic acid. Sometimes also used to describe soluble silicates or used as a synonym for soluble silicon. 

**Monosilicic acid** (MSA). Synonym: **orthosilicic acid** (OSA). MSA or Si(OH)_4_ is the simplest form of soluble silicic acid. MSA is found universally in seawater, river water and soils at a concentration of a few ppm. Although MSA is in dynamic equilibrium with disilicic acid, it is considered the only bioavailable form of silicon. 

**Oligomeric silicic acid**: disilicic acid and small clusters of monosilicic acid.

**Organic silicon**: silicon components in plant extracts and decomposed residues.

**OSA**: orthosilicic acid. Synonym: monosilicic acid. 

**PAS**: plant available silicon. Although only MSA is plant available, relevant concentrations of MSA are due to a dynamic equilibrium between monosilicic, disilicic acid and its oligomers.

**Soluble silicon**: soluble silicate salts in water including fine powdered clay particles (e.g., diatomaceous earth). Sometimes also used for dissolved silicic acid. 

**sSA**: stabilized silicic acid: monosilicic acid and its oligomers stabilized by choline (=Chol-sSA) or polyethylene glycol (=PEG-sSA) to prevent condensation/polymerization. 

**Silica (SiO_2_**): sand and rock material. Compounds: agate, amethyst, onyx, opal, sand and quartz.

**Silica colloid and gel**: aggregation of sub-colloidal silicic acid to larger colloidal particles: of colloidal silicic acid.

**Silica nanoparticles (Synonyms: Nano SiO_2_, Nano-silicon, nano silica, SiNPs)**: a synthesized amorphous silica powder consisting of silica particles with diameters at the nano size between 10–100 nm. 

**Silicates**: salts of silicic acid, a combination of silica combined with metal oxides. Used as fertilizers like calcium silicate, potassium silicate, sodium silicate and combinations of diatomaceous earth with minerals. 

### 2.2. Silicon Compounds in the Soil

There is an abundance of silicon in (most) soils. Silicon is found primarily as silicate minerals, aluminum silicates and several forms of silicon dioxide (including biogenic silica). These large quantities do not reflect the amount of soluble and plant-available monosilicic acid [16]. Due to the many variables in the soil, the conversion of these solid silicon compounds into monosilicic acid is very low [17,18]. In addition, the MSA concentration in the soil is also low due to the polymerization of MSA into oligomeric and polymeric silicic acids, resulting in a relative deficiency of monosilicic acid in the soil [19].

To overcome this MSA deficiency, several Si fertilizers are used for soil application, such as silicates, silicon dioxides sources, biogenic silica (rice hull ash, etc.) and diatomaceous earth products. All these Si products increase the MSA concentration, resulting in higher Si uptake by the plant, a higher growth rate and greater yield. These Si fertilizers have proved very effective against biotic stresses (bacteria, fungi, virus, insects and rodents) and abiotic stresses (heat, drought, acidity, salinity, etc.) [16,17,20]. 

## 3. Silicon and Plants

All plants contain Si at different concentrations according to species, ranging from <0.1 to >10% in dry weight [20]. Based on the Si content, plant species are classified as: (1) Si accumulators, with a Si content above 1.0% Si; (2) intermediate types, with a Si content between 0.5% and 1.0%; and 3) Si excluder types, with a Si content lower than 0.5% Si [21]. These differences are the result of the capacity of the roots of different plant species to absorb MSA, but within the same plant species there are also genotypic differences in Si uptake [22].

The uptake of MSA in monocotyledonous plants is an active process. Kinetic studies in rice show that the xylem loading of Si is mediated by Si transporters in the root wall. In dicotyledonous plants, the uptake of MSA is mediated by a process of diffusion resulting in significantly lower MSA concentrations in the xylem compared to monocots [23]. 

After uptake in the xylem, the (soluble) MSA follows the transpiration stream to be finally deposited in any part of the plant, within or between cells or as part of the cell wall in the case of the leaf epidermis, as silica (biogenic opal, phytoliths) [24,25]. 

Sufficient uptake of MSA by the plant is important because silicon exerts many beneficial effects on plants [26,27,28]: (a) structural strength; (b) an active role in many physiological processes e.g., a regulatory role in the uptake of other plant nutrients; (c) a role in growth and development, especially when plants are exposed to abiotic stresses (drought, salinity, acidity, etc.) and biotic stresses, because Si increases plant resistance (by stimulating defense reaction mechanism(s) [29]); and (d) decreasing damage from insects and rodents due to the fortification of the plant, acting also as a functional deterrent to herbivory.

Silicon-deprived plants are structurally weaker than silicon-enriched plants, demonstrating reduced growth, development, viability and reproduction. Moreover, these plants are more susceptible to biotic and abiotic stresses.

Today, the foliar application of (micro-) nutrients and bio-stimulants is an important method of (foliar) feeding and in some cases these applications are more effective than soil application [14,15].

The rationale for the use of foliar sprays with Silicon compounds is the assumption that foliar Si feeding could compensate for low uptake by the roots in the case of low availability of absorbable silicon in the soil, and the relatively complicated absorption process of Si by the roots, resulting in enhanced Silicon uptake with beneficial effects. 

## 4. Foliar Sprays with Silicon Compounds

Foliar sprays with Silicon compounds are classified as:

4.1. foliar sprays with silicates;
4.2. foliar sprays with (stabilized) silicic acid;
4.3. foliar sprays with other Si compounds such as: silica nanoparticles (nano-SiO_2_).

### 4.1. Foliar Silicate Sprays

Since the 1990s, foliar sprays with silicates have been used to reduce biotic stresses. 

The effects of foliar silicate sprays in reducing the infection rate of several types of pathogens are summarized in Table 1.

Foliar silicate sprays do not enhance growth or yield. Only a few publications mention that foliar silicates could have some moderate effects on growth and yield, but in the majority of the experimental trials there were other beneficial substances in the spray. 

The trials of the application of foliar sprays with silicates are classified per crop species (Section 4.1.1, Section 4.1.2, Section 4.1.3, Section 4.1.4, Section 4.1.5, Section 4.1.6, Section 4.1.7, Section 4.1.8, Section 4.1.9, Section 4.1.10, Section 4.1.11, Section 4.1.12, Section 4.1.13, Section 4.1.14, Section 4.1.15 and Section 4.1.16). The vast majority of these experiments were conducted in a randomized complete block design (RCBD) with at least three replications. In some studies, an exact specification of the concentrations used, the quantities and the spraying frequency are missing. 

Potassium silicate/meta-silicate (=K_2_SiO_3_ or KSi) is the most commonly used Si compound in the sprays, followed by sodium silicate (=Na_2_SiO_3_ or NaSi). 

#### 4.1.1. Cucumber, Muskmelon and Zucchini Squash (Study of Infection Rate)

The efficacy of root application of 1.7 mM KSi (=potassium silicate/K_2_SiO_3_) was compared with one foliar spray of KSi at two different concentrations (17 and 34 mM KSi), a spray with water plus extra K (and control), on powdery mildew of cucumber, muskmelon and zucchini squash grown in a rockwool substrate. The KSi spray (pH = 5) was applied one day before inoculation with powdery mildew conidia. Both the KSi soil application and the KSi foliar application decreased the number of powdery mildew colonies compared to the controls, showing that Si was the active agent involved [30].

#### 4.1.2. Cucumber (Two Studies of Infection Rate)

(a) In an experiment on pot-grown plants, the efficacy of root-applied 1.7 mM potassium meta-silicate (pH = 6) was compared with one foliar potassium meta-silicate spray at different concentrations of 10, 20 or 30 mM (pH = 5.5) on powdery mildew and on the production of pathogenesis-related proteins (PRs), in two cucumber cultivars with different resistance to powdery mildew. The conclusion was that foliar-applied Si can effectively control powdery mildew infections, because an effective physical barrier of Si is deposited on the leaf surfaces, and/or the osmotic effect of the applied silicates. The foliar spray did not enhance the systemic acquired resistance, while continuously root-applied Si did enhance the defense reaction through increased levels of PRs such as peroxidase, polyphenol oxidase and chitinase [31].
(b) One or two foliar KSi sprays (28 and 56 mM KSi) were applied to cucumbers plants grown on rock wool. All KSi sprays reduced the powdery mildew infection rates by up to 87% with very little difference between the two KSi concentrations [32].

#### 4.1.3. Grapes (Study of Infection Rate)

In an experiment on pot-grown plants, the efficacy of 1.7 mM KSi soil application was compared with one foliar KSi spray (17 mM) on the severity of powdery mildew of grapes. The KSi spray (pH = 5.5) was applied one day before inoculation with conidia of powdery mildew. Root-applied KSi was not effective, but the foliar spray significantly reduced the number of mildew colonies. It was shown that potassium silicates ‘coated’ the leaf cuticle, partly preventing penetration by germinating conidia. In the uncoated leaf surface areas, fungal development was more extensive [33].

#### 4.1.4. Strawberry (Study of Infection Rate)

In greenhouse and field studies, the efficacy of five foliar sprays with Ca chloride was compared with potassium silicate sprays (concentration not mentioned) on the severity of powdery mildew in susceptible varieties of strawberry plants. The KSi foliar sprays (pH = 7) reduced powdery mildew significantly. The combination with fungicide (Systhane) was more effective than either treatment alone. Calcium chloride alone was not effective [34]. 

#### 4.1.5. Rice (Three Studies of Infection Rate)

(a) In an experiment on pot-grown plants, one foliar spray at five different concentrations of potassium silicate (1, 2, 4, 8 and 16 g/L) at two different pHs (10 and 5.5) were used to control rice blast (*Pyricularia oryzae*) on a susceptible rice cultivar. The KSi spray was applied on the 22nd day after emergence (DAE). The pathogen was inoculated on the 25th DAE and disease incidence was evaluated ten days later. All KSi sprays reduced the blast incidence. The 4 g/L KSi spray appeared to be the most effective, regardless of the pH. The KSi sprays did not increase Si absorption or its accumulation by the plant [35].
(b) In an experiment on pot-grown plants, the efficacy of root-applied calcium silicate (1.25 g/kg of soil) was compared with one (40 g/L) foliar potassium silicate spray, applied one day before inoculation with the brown spot fungus at 30 day intervals and with the control (water). The foliar KSi spray decreased the intensity of brown spot, but was less effective than the root-applied Si. Only the root-applied Si induced higher Si concentrations in the rice tissue [36].
(c) In an experiment on pot-grown plants, the efficacy of three concentrations of soil-applied silica gel (60, 120 and 180 g per 5 kg soil) was compared with one sodium silicate foliar spray at three concentrations (1, 2 and 3 mM) applied one day before inoculation with rice blast fungus. All silicon treated plants showed significant increases in the Si contents of the leaves compared to non-treated plants. The silicon application reduced the disease severity significantly, with the highest reduction (75%) for the silica gel soil amendment.

The results suggest that the silicification and consequent fortification of the leaf epidermal cells mitigate disease severity. The growth parameters of the treated plants (regardless of source) did not differ significantly from the control plants [37]. 

#### 4.1.6. Soybean (Study of Infection Rate)

In a greenhouse and two field studies, the efficacy of potassium silicate sprays on soybean rust was studied. In the first two experiments, one KSi spray at different concentrations (8, 20, 40 and 60 g/L) and two pHs (10.5 and 5.5) was compared with: (a) soil application of calcium silicate; (b) foliar sprays with potassium hydroxide (KOH); (c) foliar sprays with fungicides (epoxiconazole + pyraclostrobin); and (d) control. In another field study, three foliar KSi sprays were applied at 50, 60 and 75 days after emergence. 

In the greenhouse experiment, the KSi sprays, the KOH sprays (regardless of the pH) and the fungicide sprays reduced the disease intensity of soybean rust and the number of pustules. A reduction of rust severity was also demonstrated in the field study for all KSi and KOH foliar sprays, with the highest reduction (70%) for the highest KSi rate of 60 g/L and pH = 5.5 [38].

#### 4.1.7. Wheat (Study of the Behavior of Greenbug Schizaphis Graminum)

In an experiment on pot-grown wheat plants, the efficacy of a) soil-applied calcium silicates (2.5 g/kg soil) were compared with b) the combination of soil-applied calcium silicates (2.5 g/kg soil) and foliar application of sodium silicate spray (at 5 mL/L) 15 days after plant emerge and c. the controls (untreated plants), on the biology and probing behavior of the greenbug in wheat.

All silica-treated plants showed a clear adverse effect on aphid development. Stylet penetration was not affected by the treatments, but the stylets were withdrawn more often on silica-treated plants, resulting in a reduction in the duration of probing [39].

#### 4.1.8. Wheat (Study of Infection Rate and Growth)

In an experiment on pot-grown plants, the efficacy of six weekly foliar and root applications of: (a) potassium silicate (1.7 mM); (b) stabilized silicic acid (sSA: 0.3 mM); (c) dissolved MRD-250 powder (0.9 mM Si); (d) Hoagland solution; (e) water; and (f) control (no spray), were evaluated for the reduction of powdery mildew and the growth of wheat plants. 

All root applications resulted in the deposition of Si in the leaves, with a maximum reduction of disease severity of 80%. The KSi and sSA foliar sprays reduced the powdery mildew significantly, but less than the root-applied Si. The efficacy of the MRD-250 spray was equal to the control. Soil- and foliar-applied KSi did not increase plant growth, but foliar sSA sprays showed growth-promoting effects, resulting in significantly taller plants [40].

#### 4.1.9. Poinsettia (Study of the Effect on Bract Necrosis)

In a greenhouse experiment, foliar sodium silicate sprays at three different concentrations (3.5, 5.3 and 7.1 mM), reduced the incidence and severity of bract necrosis (BN) in Poinsettias up to 30 days after initial anthesis, compared to the control. In an earlier study (1994), foliar-applied meta-silicic acid suppressed BN as effectively as NaSi (sodium silicate), indicating that silicon was the active agent [41].

#### 4.1.10. Strawberry (Study of Metabolism and Plant Growth)

Different concentrations of foliar KSi sprays (4.25, 8.5, 12.75 or 17 mM) enhanced plant growth and chlorophyll content and induced metabolic changes, such as increased citric acid and malic acid levels and decreases in fructose, glucose and sucrose contents [42]. 

#### 4.1.11. Beans (Study of Angular Leaf Spot)

In a field study, a reduction in the rate of infection of angular leaf spot in beans, resulted from four different chemical agents (sprayed three times): (a) different concentrations of KSi foliar sprays (26.7% SiO_2_ at rates of 8, 20, 40, and 60 g/L) at two different pHs (10.5 and 5.5); (b) sprays of potassium hydroxide (KOH, 13.1%); (c) sprays with fungicide (tebuconazole); and (d) control (water). The application of all KSi and KOH foliar sprays reduced the angular leaf spot severity with the greatest reduction (42%) for the KSi sprays with 60 g/L (independent of pH). The sprays with fungicide were more effective than the KSi and KOH treatments. Plant defoliation decreased with the KSi sprays. With increasing KSi concentrations, the yield increased significantly, independent of the pH [43].

#### 4.1.12. Capsicum (Study of Anthracnose, Plant Growth and Fruit Quality)

In a greenhouse, the efficacy of weekly KSi hydroponic amendments (7.5 mM) were compared with daily KSi foliar sprays (7.5 mM) and controls. Plant growth and fruit quality parameters were not significantly influenced by Si treatments, except that the firmness and cuticle thickness of the fruits were significantly higher in both soil and foliar applications. The fruits from all KSi treated plants developed significantly smaller lesions as compared to the controls. The disease incidence was delayed by several days in both KSi treatments, compared to the controls. The disease severity resulting from the Si root application was significantly lower compared to the foliar application [44].

#### 4.1.13. Mango (Two Studies of Growth, Yield and Quality Parameters)

(a) The efficacy of four potassium silicate (25% Si) foliar sprays with different concentrations (0.05%, 0.1%, 0.15% and 0.2%) was compared with four sprays of salicylic acid (100 and 200 ppm) and the control, on 18-year-old Keitt mango trees grown in sandy soil. Both types of sprays induced greater growth, yield and quality parameters. The effects of the increasing concentrations of KSi sprays showed increased growth, chlorophyll content, N, P, K and Mg concentrations in the leaves, percentage fruit set, lower physiological losses and higher yield; they were slightly more effective than the salicylic acid sprays. Combinations of KSi and salicylic sprays induced the most positive effects [45].
(b) The effects of one, two or three sprays with potassium silicate (25%) at three concentrations (0.05%, 0.1% and 0.2%) on 11-year-old Keitt mango trees, grown in sandy soil, showed significant increased growth, chlorophyll content, P, K and Mg concentrations in the leaves, percentage of initial fruit set, lower physiological losses and higher yield and quality, compared to the controls, especially when two or three KSi sprays at 0.1% or 0.2% were applied [46].

#### 4.1.14. Date Palm (Study of Growth, Yield and Quality Parameters)

The efficacy of four sprays with royal jelly (0.25–1%) and/or potassium silicate (25% Si) at three concentrations (0.05%, 0.1% and 0.2%) and/or vitamins B (thiamine 250 ppm, pyridoxine 100 ppm and cyanocobalamine 250 ppm) on growth, yield and quality parameters of 25-year-old Sakkoti date palms, grown in silty clay conditions, were studied. Single and combined applications of royal jelly, silicon and vitamins B significantly increased plant growth (total surface area per palm-leaf, total chlorophyll content), nutrients uptake of N, P, K and Mg in the leaves, yield and fruit quality. Single royal jelly sprays showed higher yields compared to silicate sprays [47].

#### 4.1.15. Grapes (Study of Growth, Yield and Quality Parameters)

The effects of four sprays with potassium silicate (25%) at three concentrations (0.05%, 0.1% and 0.2%) and/or multiple vitamins (K, E, D, A, B12, each at 50 ppm and C at 500 ppm) on growth, yield and quality of 10-year-old Flame seedless grapevines grown in clay soil were studied. KSi and vitamins sprays were effective in enhancing growth (total surface area, chlorophyll content and total carotenoids), nutrient uptake of N, P, K, Mg, Zn and Fe in the leaves, and fruit yield and quality. The results for the sprays with vitamins were superior to those of silicate sprays. Combined application was more effective on yield (clusters/vine and yield/vine) and fruit quality [48]. 

#### 4.1.16. Chili (Study of Growth, Yield, Quality Parameters and Infection Rate)

Application of three KSi (18% Si) sprays (1, 2 or 4 mL/L), combined with Zn (0.2%) and B (0.1%) starting 30 days after transplant at two-week intervals increased plant growth, nutrient uptake and yield, especially for the 4 mL/L KSi spray. Disease incidence from powdery mildew and leaf spot was significantly lower compared to the control [49]. 

### 4.2. Foliar Sprays with (Stabilized) Silicic Acid (sSA)

Up to the end of last millennium, silicic acid was not available on the market because of its instability. Since 2002, mono-silicic acid, the only plant-available silicon form, can be used due to patented production processes in which the polymerization of silicic acid is prevented [50]. After 2003, many trials were conducted to test the efficacy of this direct form of plant-available Si. Because bioavailable silicic acid is used, the concentration of SA in the foliar sSA sprays is higher compared to that available in the soil.

Several silicic acid products can be classified according to the stabilizing agent used: a) choline (=Chol-sSA) or b) polyethylene glycol (=PEG-sSA). Both categories of sSA products contain concentrated forms of SA (2–2.5%), with a mere Si content of 0.7–0.8%. Both sSA products are very acidic in order to prevent polymerization. These concentrated products have to be greatly diluted 1000 to 170 times (=1–6 mL/L) with water before being used as a foliar spray (with Si concentrations ranging from 7 to 45 ppm). After dilution, the pH ranges between pH 4–7. For optimal use, this is adjusted to pH = ±5.5. In most studies a wetting agent is added.

In the first experiments with sSA on lettuce, onions, potatoes, flowers and fruit trees, during 2001–2003, the optimal concentration of sSA in the sprays and the optimal spraying frequency for most crops were determined to be 2–4 mL sSA/liter of water and a quantity of 150–400 L spray/ha. For most crops, the optimal spraying frequency is 3–4 sprays with ± two-week intervals starting early in the vegetative stage, when 3–5 leaves have appeared [19]. Other crops, like sugarcane and fruit trees, need other spraying regimes. 

The relevant trials since 2003 with different sSA sprays on different crops are summarized below (Section 4.2.1, Section 4.2.2, Section 4.2.3, Section 4.2.4, Section 4.2.5, Section 4.2.6, Section 4.2.7, Section 4.2.8, Section 4.2.9, Section 4.2.10, Section 4.2.11, Section 4.2.12, Section 4.2.13, Section 4.2.14, Section 4.2.15 and Section 4.2.16).

If not stated otherwise, the trials were carried out in open field studies according to a RCBD (randomized complete block design) with at least three replications. 

#### 4.2.1. Potato (Five Studies of Growth, Yield and Infection Rate)

(a) The application of eight sprays with 4 mL/L PEG-sSA (30 ppm Si) on potatoes grown in clay soil in the Netherlands effected greater disease resistance against Phytophthora and a yield increase of 6.2% compared to the controls and an improved the proportion of large grade (size) tubers [51].
(b) The efficacy of weekly sprays with 5 mL/L silicic acid (38 ppm Si; type of SA is not mentioned) in a field study in Brazil did not show any influence on plant development, productivity and the incidence of beetles and aphids [52].
(c) The efficacy of four sprays with 2 mL/L PEG-sSA (15 ppm Si) was studied on the yield, quality and insect infestation on three different potato varieties at three different field locations in Brazil. The PEG-sSA applications increased tuber weight by 39.6%, 14.2% and 0.1% according to variety, also seen in an increased tuber dry matter content, a reduction in the severity of late blight and the incidence of blackleg [53].
(d) In an experiment on pot-grown plants, the effect of three sprays of PEG-sSA (35 ppm Si) at 10, 20, and 30 days after emergence, were compared with the soil application of silicates on the Si content, nutrient uptake and pigment concentration, as well as the gas exchange and growth of potato plants. Foliar sprays and soil application increased the Si accumulation in the whole plant. Foliar spraying resulted in the highest Si concentration in leaves, while soil amendment increased the Si accumulation in roots, stems and leaves. Both soil application and foliar PEG-sSA sprays increased the leaf area and pigment concentration (chlorophyll *a* and carotenoids), as well as the photosynthesis and transpiration rates of the potato plants [54].
(e) The effects of 1, 2 and 4 mL/L sprays with PEG-sSA (8–32 ppm Si) on three potato varieties showed in every case yield increases of up to 20%. Leaf size, chlorophyll content and nutrient status were improved significantly. Also, the dipping of the tuber in 1 mL/L PEG-sSA increased the tuber yield. The incidence of leaf blight was also reduced [55].

#### 4.2.2. Onion (Study of Yield)

The efficacy of four sprays of 4 mL/L (30 ppm Si) PEG-sSA was studied on two different onion cultivars grown in clay soil in The Netherlands. The outcome showed an increase of +9.9% and +10.8%, respectively [51]. 

#### 4.2.3. Papaya (Study of Growth, Yield, Quality and Infection Rate) 

The efficacy of four sprays of 4 mL/L (30 ppm Si) PEG-sSA was studied in Colombia, using two different spraying regimes compared to the control. The PEG-sSA treatments showed an increase in plant height (+6.3 and +7.8%), stem diameter (+7.3 and +8.2%), an increase in fruit weight (+11.9% and +13.2%). The flavor of the fruits was superior to those of the control plants. There was a significant disease reduction of smallpox (Asperisporium caricae) [56]. 

#### 4.2.4. Rice (Three Studies of Growth, Yield and Infection Rate)

(a) The efficacy of four sprays of 4 mL/L (30 ppm Si) PEG-sSA was studied on the growth, yield and infection rate of rice in Panama. The growth and yield parameters increased. The rice-ears carried more and larger grains. Yield increased by 9.6%. The crop cycle was one week shorter compared to the control [57].
(b) The efficacy of four sprays of PEG-sSA with low-dose boric acid was studied in the field using seven treatments of 2, 4 and 8 mL PEG-sSA/L (15, 30 and 60 ppm Si) plus different dosages of insecticides (Monocrotophos) and fungicides (Carbendazim), for two years at two different field locations (sandy loam and sandy clay loam soils). The results revealed a significantly higher grain and straw yield compared to the control plants. Foliar PEG-sSA sprays (2 and 4 mL/L) increased all growth parameters, resulting in a maximum grain yield of +32%. Foliar 4 mL/L PEG-sSA sprays resulted in the highest straw and grain yields and Si content, when the pesticides rates were reduced by 50% compared to the control plants [58,59].
(c) PEG-sSA (2 mL/L) applied once only to seedlings induced a significantly higher chlorophyll content and an enhanced uptake of nutrients (P, Ca and K) compared to control plants. In a field study, three sprays of PEG-sSA (2 mL/L) with two-week intervals were applied. Foliar PEG-sSA increased growth parameters (root volume, number of tillers) and yield parameters significantly. Moreover, the infection rate of the sprayed plants was reduced by 70%; the number of white ear-heads was reduced from 10.3/m^2^ in control plots to 4.3/m^2^ in the sprayed plots [60].

#### 4.2.5. Strawberry (Study of Growth and Yield)

The efficacy of three sprays with 0.1% and 0.2% Chol-sSA (7 and 14 ppm Si) on the growth parameters of strawberry plants grown in different soil types, showed that both concentrations significantly improved growth (root length, root mass weight, diameter of root neck, leaf blade area) in all soil types [61]. 

#### 4.2.6. Grapes (Two Studies of Growth, Yield and Quality)

(a) The effects of four sprays with 2, 4 and 6 mL/L (15, 30 and 45 ppm Si) of PEG-sSA at different dose rates on 18-year-old plants of Bangalore blue grapes, grown in red sandy loam soil (pH 5.5), showed that all growth parameters (cane length, leaf area and total chlorophyll content) increased significantly. The number of bunches per vine, yield per vine and yield per hectare were significantly increased under all PEG-sSA spray regimes, especially in the 4 and 6 mL sprays, which resulted in a max yield increase of +39%. The uptake and accumulation of nutrients (K, Ca, B, P and Si) in the petiole was significantly higher in all foliar SA treatments compared to the control (water). Quality parameters such as total soluble solids, acidity, total sugar, reducing sugars, non-reducing sugars, physiological loss in bunch weight and the percentage of rotten berries, were positively influenced [62].
(b) The efficacy of one to four sprays with 1, 2 and 3 mL/L (=8, 15 and 23 ppm Si) of PEG-sSA at different dose rates on plants of Thompson seedless grapes, over two years, showed an increase in berry length and diameter, increases in bunch weight and improved berry quality. The maximum bunch weight resulted from four PEG-sSA sprays (3 mL/L) [63].

#### 4.2.7. Finger Millet (Study of Growth, Yield and Infection Rate)

The effects were studied of two sprays of 2 and 4 mL/L (15 and 30 ppm Si) PEG-sSA on 10 different finger millet varieties (with different genotypic accumulation of Si) on growth, nutrient uptake and resistance to blast disease. In all genotypes, the highest Si accumulation was found first in the glumes and then in the straw and grains. The grain and straw yield increased significantly, with the greatest increase at the 4 mL/L PEG-sSA rate, while the Si uptake increased 54.6% over the control. The 2 and 4 mL/L sSA sprays reduced the blast disease by 50.4–69.8% in the different genotypes [64]. 

#### 4.2.8. Sugarcane (Three Studies of Growth, Yield, Quality and Infection Rate)

(a) In India, 2010–2012, the efficacy of six and twelve sprays with 4 and 6 mL/L (30 and 45 ppm Si) PEG-sSA on growth and yield was compared with a) soil application of calcium silicate and b) the combination of soil application of calcium silicate and foliar PEG-sSA application. All foliar PEG-sSA sprays resulted in increased growth (cane length, cane girth, number and length of internodes) and yield parameters (number of millable canes, single cane weight and cane yield), compared to the soil application of calcium silicate at 500 kg/ha. The 4 mL sprays increased the yield +26%, being more effective than the 6 mL sprays, while the soil application resulted in a +14% yield increase. The maximum yield was obtained by the combination of soil and leaf application, namely +33% [19].
(b) The effects of one aerial spray with PEG-sSA was compared to a spray with a combination of glyphosate and sodium metasilicate (2%) on two sugarcane varieties. The PEG-sSA spray increased the cane yield by 4.6%, while the average cane weight decreased by 3.9% as a result of the glyphosate/metasilicate application. Both PEG-sSA and glyphosate/metasilicate treatments showed increased Brix values (sugars) and juice purity compared to the control [65].
(c) The effects were studied of one to four PEG-sSA sprays with concentrations of 1, 2, 3 and 4 mL/L (7, 15, 22 and 30 ppm Si) on yellow mite *Oligonychus sacchari* *McGregor*, on two sugarcane varieties, over a two-year period. All treatments significantly decreased the mite population and leaf dryness compared to the control. Four 4 mL/L PEG-sSA sprays appeared to be the most effective [66].

#### 4.2.9. Ornamental Plants (Study of Growth, Yield and Quality)

The effects of three sprays with 2 and 3 mL (15 and 22 ppm) Chol-sSA on seasonal ornamental plants (e.g., Marguerite daisy (*Argyranthemum frutescens*), strawflower (*Xerochrysum bracteatum*), African daisy (*Osteospermum ecklonis*) and Gaura (*Gaura lindheimeri*) grown in peat and peat-sand substrate. The results showed an increase in the number of lateral shoots in all ornamental plant varieties in relation to the increase in product concentration. All Chol-sSA sprays increased the number of buds and flowers or inflorescences, with an increased flower diameter also dependent on the concentration of the spray [67]. 

#### 4.2.10. White Oat and Wheat (Two Studies of Growth, Yield and Quality)

(a) Three foliar sprays with 2 mL/L (15 ppm Si) PEG-sSA increased N, P, K and Si concentrations in the flag leaves of white oat, resulting in higher shoot dry matter. The number of panicles, number of grains per panicle and overall grain yield increased significantly.
  In wheat, the PEG-sSA sprays increased the K and Si concentrations in the flag leaves, the weight of the shoot dry matter and the number of spikes per m^2^, resulting in an increase in grain yield of 26.9% [68].
(b) The effects of three PEG-sSA foliar sprays at concentrations of 0.5, 1 and 2 mL/L (4, 7 and 15 ppm Si) were evaluated for alleviating drought stress in wheat. The 2 mL/L sSA sprays showed the greatest efficacy in terms of the increase in the relative water content of the plant, leaf chlorophyll content and lower canopy temperatures. Growth parameters, such as root growth and root length, and quality parameters, such as K and P content in the straw and seed, increased. The yield (seed weight) increased significantly (>10%) under drought stress [69].

#### 4.2.11. Soybean, Common Bean and Peanut (Two Studies of Growth, Yield and Quality)

(a) Four sprays of 2 mL/L (15 ppm) PEG-sSA were applied to plants of soybean, common bean and peanut. The foliar PEG-sSA sprays increased the pod numbers and seed yields: 14% for soybean, 15% for common bean and 9.6% for peanuts [70].
(b) Two and three PEG-sSA sprays with concentrations of 2 or 4 mL/L (15 or 30 ppm Si) on two cultivars of soybean increased the plant height, number of leaves, pod and seed yield. The protein and oil yield increased significantly. Overall, three sprays with 2 mL/L PEG-sSA was the most effective foliar application for soybean [71].

#### 4.2.12. Tomato (Two Studies of Growth, Yield, Quality and Infection Rate)

(a) The efficacy of three foliar PEG-sSA sprays of 4 mL/L (30 ppm Si) on the early growth and the composition of mineral elements in tomato transplants was studied. The sprayed tomato transplants were taller with larger stem diameters. The NO_3_, N, P, K and Ca concentrations were enhanced [72].
(b) The efficacy of three foliar PEG-sSA sprays of 2 and 4 mL/L (15 and 30 ppm Si) on the reduction of powdery mildew disease in tomato was compared with standard fungicide (Difenoconazole) and the control. In this pot experiment, the 2 mL/L PEG-sSA spray reduced disease severity by 26% and the 4 mL/L sSA spray by 56% compared to the control. The 4 mL/L PEG-sSA sprays resulted in a greater reduction in disease severity compared to the fungicide control.

The highest dry weight and silicon content in the leaf resulted from the 4 mL/L PEG-sSA sprays [73].

#### 4.2.13. Maize (Study of Growth and Yield)

The efficacy of three PEG-sSA sprays at concentrations of 1, 2 or 3 mL/L (7, 15 and 22 ppm Si), applied to maize plants at 20 days intervals, was compared with the application of silicon granules to plants growing in sandy loam and clay loam soils. Both soil and leaf applications enhanced the growth and yield parameters in both soil types. The highest yield resulted from the combination of the foliar PEG-sSA application (3 mL/L) with the soil application of silicon granules [74]. 

#### 4.2.14. Mango (Study of Growth, Yield and Quality)

The efficacy of four 4 mL/L (30 ppm Si) PEG-sSA sprays applied at three-week intervals was compared with: (a) four silicate sprays; (b) soil application of calcium silicate; (c) rice husk ash (RHA); and (d) control. All soil and foliar silicon applications enhanced the growth and yield. Calcium silicate was more effective than RHA. The PEG-sSA foliar sprays were more effective in terms of yield increase and TSS (total soluble solids) compared to the silicate sprays and silicon soil applications. The shelf life of the fruits from the PEG-sSA treated trees increased by five days compared to the application of other types of silicon-containing products [75]. 

#### 4.2.15. Mandarin (Study of Yield and Quality)

The efficacy of one, two and three PEG-sSA sprays of 1, 2 or 4 mL/L (7, 15 and 30 ppm Si) at 30 days interval, was studied in Mandarin plants with fruit at the pea stage and ripening stages. Sprays with 2 mL sSA at the pea stage enhanced the nutrient status of leaves and fruits and resulted in an increase in fruit size of +25%. Application during the ripening stage enhanced the volume of juice, especially with the 4 mL/L PEG-sSA foliar sprays. The layers of biogenic silicon in the rind of the fruits increased, as did the sugar content of the juice, together with better nutrient ionic status. The shelf life of the fruit also increased [76]. 

#### 4.2.16. Other Crops

An overview of other results for the foliar application of PEG-sSA is provided in Table 2. Most of these results were published in 2016 [19]. The field trials used 2–4 mL/L (15 and 30 ppm Si) PEG-sSA. All trials showed increases in growth, yield and quality parameters resulting from a reduction of the biotic and abiotic stresses.

**Figure 1:**
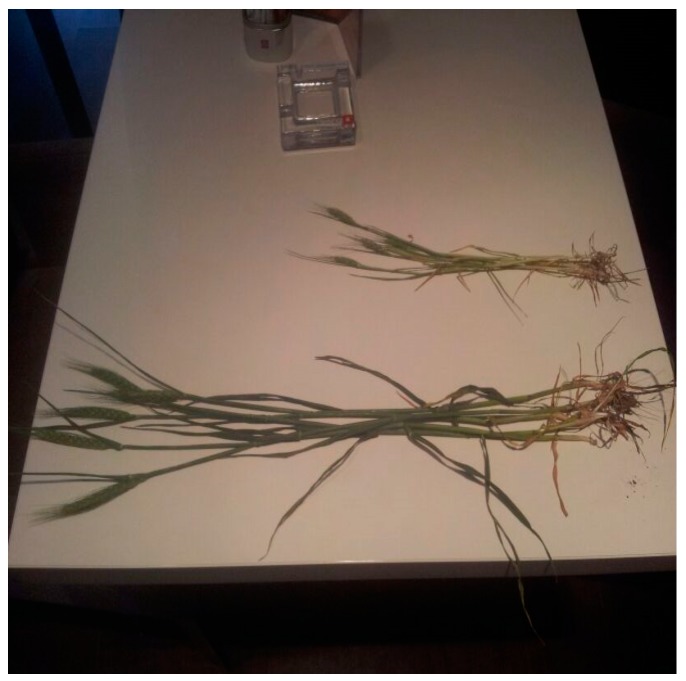
The effects of foliar application of PEG-sSA on wheat grown in very saline soil (area of 3 ha) in Romania during 2014. Half of the plot was sprayed four times with AB Yellow^®^ (=4 mL/L PEG-sSA plus B, Mo and Zn). The visual differences between the sprayed plants (taller plants) and the control (small plants) can be seen.

**Figure 2:**
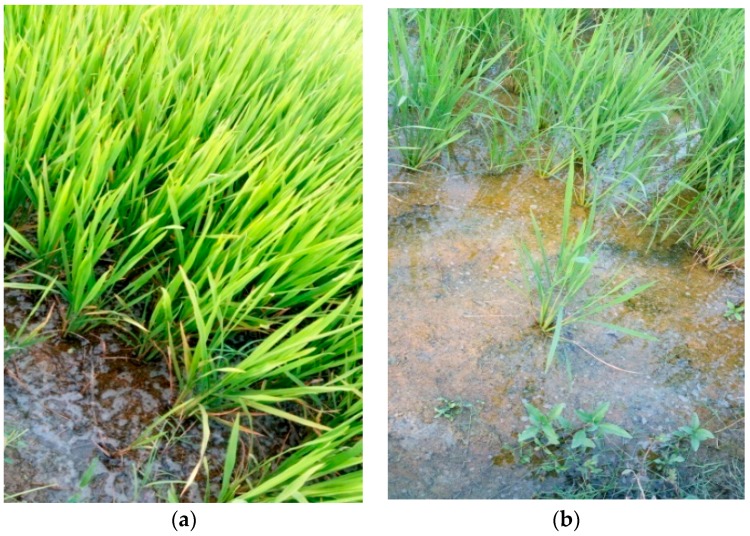
Rice plants grown on very acidic soil on a plot of 1.5 ha near Chennai (India), 2017. Half of the plot was sprayed with PEG-sSA. The visual differences between sprayed plants (**a**) and the control (**b**) can be seen.

The effects of the application of PEG-sSA can be seen in Figure 1 (increased length of tillers of wheat grown on very saline soil) and the effects of the application of PEG-sSA on rice grown on acidic soil compared to traditional agricultural practices (Figure 2a,b).

### 4.3. Other Foliar Silicon Compounds

Other silicon sources for use in foliar spraying are silica nanoparticles (synonyms: nano-Si, silicon dioxide nanoparticles, SiNPs) and Diatomaceous earth (DE). 

Silica Nanoparticles

Silica nanotechnology is the engineering and use of nanosized particles of silica as an antifungal agent, biopesticide and agro-fertilizer. Silica nanoparticles (nano silica) are produced from several sources, like tetraethylorthosilicate (Si(OC_2_H_5_)_4_), or inorganic salts, such as sodium silicate (Na_2_SiO_3_). Another (organic) source for silica nano-particles is rice husk. 

These synthesized silica powders are amorphous with diameters in the range of 10–100 nm. Silica nanoparticles with a size from 20 to 40 nm are most commonly used in foliar sprays.

#### 4.3.1. Bamboo (Two Studies of the Effect on Physiology)

(a) In a field study, one foliar spray (nano-Si concentrations: 0, 150, 300 and 450 mg/L) on Indocalamus barbatus McClure showed that all nano-Si sprays increased the gas exchange parameters and chlorophyll parameters, such as photosynthetic rate, transpiration rate, stomatal conductance, photochemical efficiency, etc. The most effective concentration was 300 mg/L nano-silica [77].
(b) In a field study, one foliar spray (nano-Si concentrations: 0, 150, 300 and 450 mg/L) on bamboo showed that all concentrations of nano-Si treatment increased the protein contents, free amino acids, total nitrogen, phosphorus, potassium, stimulate SOD (superoxide dismutase) and POD (peroxidase) activities, and decreased the malondialdehyde (MDA) content. Among the different concentrations of Nano-Si foliar sprays, 300 mg/L appeared to be the most effective spray for increasing the nutritional status and the ability to scavenge active oxygen [78].

#### 4.3.2. Maize (Study of Physiology and Safety)

In a pot study, the effects of one foliar spray with silica nanoparticles (20–40 nm) were compared with: (a) one spray with KSi (15 g/L); (b) soil application of silicates; and c) control (no spray) on the phytochemical responses of maize. The results of the study showed significantly higher concentrations of organic compounds and silica contents in the nano-silica treatment compared to the silicate spray and to control. On the other hand, the soil application of silicates appeared to be more effective than the foliar nano-Si sprays [79].

#### 4.3.3. Safflower (Study of Growth and Yield)

The efficacy of three foliar (20 mM) nano-Si sprays (particle size: 20 to 30 nm), applied at the leaf development, branching and capitulum emergence stages, were studied on growth and yield. Four fertilizer regimes were used: (a) application of 30 t/ha farmyard manure (FYM); (b) application of 15 t/ha farmyard manure (FYM); (c) application of 90 kg/ha NPK fertilizers; and (d) control (no fertilizers). Although the nano-Si sprays significantly improved some growth parameters such as canopy spread, stem diameter, plant height, ground cover and the number of achenes in the capitulum, there was no effect on the achene yield or the harvest index [80]. 

#### 4.3.4. Jatropha Integerrima (Study of Salt Stress)

In an open field experiment, the effects of eight silica nano sprays (1 or 2 mM) and gypsum (calcium silicate) soil application on the growth, flowering and chemical constituents of Jatropha integerrima plants under different levels of saline water, were studied. The results showed that foliar nano-Si sprays and the soil application of gypsum, either individually or in combination, enhanced the growth parameters and chemical constitutes (such as chlorophyll) and decreased the accumulation of Na, Cl, phenolic compounds and flavonoids in the leaves in case of salinity (up to 4000 ppm) [81].

#### 4.3.5. Cucumber (Study of Effects of Salt Stress and Growth)

In a field study the efficacy of one nano-Si spray with different concentrations (0, 15, 30, 60 and 120 mg/L) on the growth, yield and chemical composition of cucumber was studied. The nano-Si spray increased the growth parameters (plant height (cm), number of leaves/plant, fresh and dry weight of leaves/plant (g), number of fruits/plant, mean weight of fruit, fruit length) and yield parameters (kg/plant, total yield t/ha) compared to untreated plants. The 60 mg/L nano-Si spray showed the highest increase for most growth parameters [82].

#### 4.3.6. Squash (Study of the Mortality Rate of the African Cotton Leafworm)

The efficacy of two nano-Si sprays (with different concentrations of 200, 300, 400 and 500 ppm Si) were compared with (a) silicate sprays; (b) Diazinon (standard recommended insecticide); and (c) water (control) on newly hatched Spodoptera littoralis larvae on squash plants in a greenhouse. The neonate larvae mortality rate was 68% for the nano-Si spray at 500 ppm; 6.8% for silicates, and 51.6% for Diazinon and no mortality with distilled water. The lifespan of the PEG-sSA treated plants was longer compared to all other treatments [83].

*Diatomaceous Earth/Other Silicon Solutes*

There is very limited information about the efficacy and safety of the use of DE and other silicon solutes as foliar spray. Because most of these products also contain other beneficial elements (such as Ca, Mg and Iron), the effectiveness of silicon in these sprays cannot be properly determined. For this reason, these sprays are not considered further in this review. 

## 5. Discussion

From the 1990s, foliar sprays with silicon have been used. Three different types of Silicon compounds have been used: sprays with silicates, sprays with (stabilized) silicic acid and sprays with silica nanoparticles (SiNPs). Each type of silicon compound in the foliar spray shows specific characteristics. 

*Foliar Sprays with Silicates*

Foliar sprays with silicates reduce infections of powdery mildew on cucumber, muskmelon and zucchini [27,28], grapes [33], strawberries [34] and wheat [40]. Similar effects were found for the reduction of the incidence of rice blast [35], brown spot [36], soybean rust [38], angular leaf spot in beans [43] and anthracnose in capsicum [44]. A reduction in greenbug infestations in wheat was also found [39] (Table 1). These spray(s) do not enhance plant growth or yield, especially at higher concentrations. In a few trials using lower concentrations of KSi, some positive effects on plant growth and development were reported. This could be an indirect effect of the relationship between a lower infection rate and greater plant growth. Moreover, in most of these studies, the sprays also contain other beneficial substances (such as vitamins), which could also have stimulating effects on growth [47,48]. 

Based on all studies, *repeated* foliar applications of potassium silicate appeared to be more effective than a *single* spray. The higher concentrations resulted in the greatest reduction of infection [38].

When the efficacy of foliar-applied silicates is compared with soil-applied silicon fertilizers, the silicate sprays appeared to be less effective. This can be explained by observing that foliar silicate sprays do not enhance systemic acquired resistance, while soil-applied Si sources do [31]. 

In a comparative study of beans, the application of foliar silicate sprays was compared with fungicide (tebuconazole). The fungicide sprays appeared to be more effective than the KSi foliar treatments [40,43]. On the other hand, silicate sprays are safer for living organisms and have a lower (negative) impact on the environment. 

The mode of action of KSi sprays is not fully understood. Foliar-applied silicates induce a higher Si content in silica cells together with a significant increase in the Si content of the leaf [37]. Other trials showed that foliar-applied KSi induced a significant reduction in fungal proliferation, demonstrating that potassium silicate exerts a suppressing effect on the fungi on the leaf surface [31]. Foliar sprays with KSi enhance passive immunity by forming a physical barrier on the leaf surface (silicification), while osmotic effects partly prevent pathogens from penetrating the leaf epidermis [31].

Overall it can be concluded that foliar sprays with silicates can be used as an environmentally safe pesticide due to the formation of an effective physical barrier of Si, deposited on leaf surfaces, and due to the osmotic effect of the applied silicates. 

There is no direct effect on growth and development.

*Foliar Sprays with (Stabilized) Silicic Acid*

Monosilicic acid (MSA/OSA) is supposed to be the only plant-available silicon compound. However, in wheat, the transport of Si through the plant was studied using a Si solution containing silicic acid with a ^29^Si radioactive isotope. Xylem exudate was collected and analyzed for Si using nuclear magnetic resonance (NMR). Two Si species, mono- and disilicic acid, were found [84], showing the extreme propensity of MSA to polymerize; monosilicic acid is always in dynamic equilibrium with disilicic acid. 

Some commercial producers of stabilized silicic acid claim that their product contains 100% monosilicic acid, but this is incorrect. Commercial products with stabilized silicic acid contain monosilicic acid as well as its oligomers (disilicic acid, trisilicic acid, etc.), because in an aqueous solution monosilicic acid is in equilibrium with its oligomers. 

*Efficacy of Foliar Sprays with Stabilized Silicic Acid Compared to Foliar Silicate Sprays and Soil-Applied Silicon Fertilizers*

Foliar sprays with sSA increased growth, yield and quality parameters, especially when stress factors are involved. 

In a comparative study of wheat with (a) foliar-applied silicates; (b) soil-applied silicates; and (c) foliar-applied PEG-sSA, only the sSA treated plants showed growth-stimulating effects, such as significantly taller plants [40]).

The effects of the sSA sprays can be compared with the effects of soil amendments with Si fertilizers. With the former (sSA), trials have demonstrated increased root mass [57], more and thicker tillers [55,56,57], and larger leaf surfaces with higher chlorophyll content [59]. Further trials recorded: increases in quality, measured by the higher nutrient content of the plant [59], increased sugar content (sugarcane) and increased lycopene and ascorbic acid (tomato), etc. [19]. Moreover, sSA sprays are equally effective for monocots as for dicots, while soil applied silicon amendments are hardly or less effective for dicots.

In a study on sugarcane, the efficacy of foliar PEG-sSA was compared with soil-applied calcium silicates. sSA sprays appeared to be more effective on growth and yield parameters compared to the soil application of calcium silicate [19]. 

In a comparative study of wheat between foliar silicates, foliar sSA and soil-applied Si, only the foliar sSA treated plants showed an increase in growth [40].

*Efficacy of Foliar Sprays with Stabilized Silicic Acid on the Infection Rate*

sSA sprays significantly reduce many bacterial and fungal infections [19], allowing a reduced use of pesticides, with at least 50% compared to the control [59,73].

In conclusion, the results showed that foliar sprays with sSA could be used as an ecofriendly alternative to the pesticides effective for managing different diseases (fungi, virus, etc.).

*Spraying Regimen and Concentration of Stabilized Silicic Acid Sprays*

The sSA sprays are only effective if applied at the (early) vegetative stage. The efficacy is further increased by using several sprays (3–4) at an interval of 10–20 days, dependent on plant species.

The concentration of Si in the sSA sprays was 7–45 ppm (1–6 mL of the product with ±2.5% sSA/L). These concentrations are 20–100 times lower compared to the Si concentration in foliar silicate sprays (500–1000 ppm). 

The optimal sSA concentration of the spray differs according to the crop. For growth stimulation, 2 mL/L silicic acid is sufficient for most crops, while 4–6 mL is optimal for sugarcane. The best results in reducing infection rates and mite infestations are achieved using concentrations of 4 mL/L sSA [64,66]. Concentrations of silicic acid >6 mL/L (60 ppm) are not effective; no increase of growth and yield were obtained compared to the control [56].

A spraying frequency of 3–4 times is optimal for most crops; 3–4 sprays are more effective than one or two.

In conclusion, sSA sprays are only effective when concentrations of 1–6 mL/L (Si: 7–45 ppm) are used at 10–20-day intervals, from the early vegetative stage onwards. Positive effects on growth, development, quality and the reduction of infections and mite infestations have been reported for plants grown in alkaline, neutral and more acidic soils, especially when stress factors such as salinity and acidity are involved (Figure 1 and Figure 2) and drought stress [69].

*Mode of Action of Foliar-Applied sSA*

The first effect of foliar sSA application is enhanced root growth (compared to the control) as can be seen in rice [57] and other crops. Other growth parameters are also increased, such as the number of tillers, plant height, panicle length and leaf surface area [59]. The enlarged root system allows an increased uptake of nutrients. In many plant species, foliar sSA applications increase the nutrient uptake as measured by yield (e.g., grain) and straw, the concentrations of N, P, K and Si in the flag leaves [68] and the concentrations of P, Ca and K in young rice plants [60]. In grapes, the uptake and accumulation of nutrients (K, Ca, B, P and Si) in the petioles were significantly higher in sSA foliar treatments, compared to the control [62]. In addition, the concentrations of N, P, K and Ca were enhanced in sSA-sprayed tomato plants [72].

The growth-stimulating effects of foliar-applied sSA can be compared with the soil application of silicates [26,27,28]. The efficacy of silicate amendments is the result of the release of monosilicic acid, which is the biologically active substance. On the other hand, to be sufficiently effective, soil application requires large quantities of silicate fertilizers (1–3 tons/ha), because only a very small fraction of the silicates is converted into the bioavailable monosilicic acid, while sSA sprays contain monosilicic itself (or its oligomers), so only 3 L/ha of a commercial product with sSA is needed in the foliar sprays for one crop cycle. 

Despite the many positive effects of foliar sSA, the exact mode of action is not understood. Although it has been established that plant leaves and other aboveground parts are capable of absorbing all kinds of chemicals and (micro-) nutrients, the uptake/absorption mechanism of monosilicic acid by the leaves has not been established. On the other hand, in studies with foliar sSA versus controls (water or water with potassium or water with PEG), only sSA appeared to be effective. Therefore, the absorption of sSA by leaves is most likely. The assumption that the effects of the foliar application of sSA are caused by its run-off to the soil and subsequent uptake by the roots, is disproven by experiments where the soil surface around the plants was covered with plastic (polyethylene sheeting).

Further research is needed to prove this assumption; the ways in which SA is able to pass through the leaf cuticle or stomata into underlying cells. Additionally, it is necessary to discover the mechanism behind the way MSA is able to translocate between cells (through cell walls and intercellular spaces) and in what ways MSA influences plant physiology. 

In the roots of monocots, the uptake of Si is facilitated by membrane transporters [21,22], while in dicots the uptake takes place by (passive) diffusion, in the absence of membrane transporters [23], which results in significantly lower MSA concentrations in the xylem. In case of foliar-applied sSA, the efficacy of Si on plant growth and development is the same in monocots *and* dicots.

The effects of foliar-applied sSA show similarities to the foliar application of salicylic acid, such as the increase in growth, yield and quality parameters [85]. Moreover, there are similarities with the effects of other plant hormones such as gibberellic acid (GA), jasmonate (JA), auxin and cytokines. For example, GA stimulates seed germination and is involved in the interactions with environmental factors such as light, temperature and water. Stabilized silicic acid and GA show similar effects on seed germination. Overnight soaking of rice seeds in water with 0.1% sSA increases germination and root development significantly. In addition, the effects of this seed treatment with sSA are seen in seedlings (40 days after transplanting) with greater root mass, longer shoots and more tillers, with >50% increases in the dry weight of roots and shoots [60]. 

The efficacy of foliar sSA is relatively higher in crops grown in suboptimal soils and under suboptimal conditions, showing similarity with the role of jasmonate in inducing plant responses to poor environmental conditions and plant defense mechanisms.

Based on all these effects, the hypothesis can be postulated that sSA is absorbed by the leaf and fulfills a role as a signaling molecule for the activation of growth-promoting and anti-stress hormones. 

*Safety of (Stabilized) Silicic Acid*

Stabilized silicic acid is used as an oral food supplement for humans and animals [50] in almost the same concentrations. Silicic acid is (relatively) safe; Chol-sSA and PEG-sSA are registered for human consumption and these compounds are known to be non-toxic [86].

The sSA sprays are safe for the plant, soil, farmer, surface water and groundwater, insects and consumers.

*Classification of Foliar-Applied Stabilized Silicic Acid*

Foliar sSA should be classified as a biostimulant. The effects of foliar-applied sSA show similarities with (some) other foliar-applied biostimulants like seaweed-derived sprays and biostimulants derived from microbial fermentation. Both biostimulants increase shoot length, cause larger leaf area and more biomass [87], similar to the effects of sSA sprays on rice [58,59] and other crops. Like the sSA sprays, the mode(s) of action of these biostimulants is also not clear.

*Foliar Sprays with Silica Nanoparticles*

To date, only limited data on efficacy have been published. The results of studies so far on silica-nano feeding show positive effects on growth and yield, especially when higher concentrations are used. In other studies, a (modest) reduction of the effects of biotic stresses was obtained. 

When the efficacy of nano-silica sprays is compared with foliar-applied sSA sprays, the former appears to be less effective with respect to growth and yield parameters and in reducing the infection rate. Compared to the soil application of silicates, the foliar-applied silica nanoparticles appeared to be less effective [79]. On the other hand, in a comparative study with fenugreek (*Trigonella foenum-graecum* L.), between soil applications of silicates versus silicon nano-particles on Si accumulation, the activity of several antioxidative stress enzymes, and the lignification of xylem cell walls, Si uptake from both soil amendments showed no difference in effectiveness [88]. This could be explained by the fact that soil-applied SiNPs are more effective than foliar-applied silica nanoparticles.

The mechanism of action of foliar SiNPs has not yet been clarified. Due to the nature of these particles, made up of several thousand up to millions of silicon dioxide units, it is likely that a (small) part of the SiO_2_ particles are converted into (bioactive) MSA molecules. In addition, it is likely that a mechanical effect of the nanoparticles causes a significant increase in the mortality rate of neonate *Spodoptera littoralis* larvae (African cotton leafworm) [83].

## 6. Summary and Conclusions

Foliar sprays with silicon compounds can be divided into sprays with: (a) silicates; (b) stabilized silicic acid; and (c) silica nanoparticles. 

The effects of these three types of sprays are summarized in Table 3.

Foliar sprays with silicates are effective as pesticides. There is no systemic effect on plant growth, development and yield.

Foliar sprays with stabilized silicic acid enhance root and plant growth, yield and quality in monocots as well as dicots in almost any soil type. These sprays are very effective against biotic and abiotic stresses. 

Sprays with silica nanoparticles have some positive effects on growth and yield and are capable of decreasing the infection rate.

The sSA and nano-SiO_2_ sprays can be classified as ‘biostimulants’. These sprays enhance nutrient uptake and nutritional efficacy and decrease abiotic and biotic stresses resulting in the stimulation of growth and yield increases. The use of sSA sprays allows a significant reduction in the use of pesticides without losing efficacy. Moreover, the sprays with stabilized silicic acid are safe for the plant and the environment. In view of these results, sSA deserves much more attention as an ecofriendly alternative to pesticides.

As sSA sprays are very effective against abiotic and biotic stresses, foliar sSA sprays should be used as an ‘insurance policy’ in suboptimal conditions, created for example by climate change and fluctuations in environmental conditions.

Due to all these positive effects, sSA sprays should be considered a reliable, safe and effective alternative to GMO crops.

## Figures and Tables

**Table 1:** Summary of the efficacy of foliar silicates sprays (KSi: potassium silicate; NaSi: sodium silicate) on the infection rate of several common diseases in monocots (M) and dicots (D). In separate columns: product concentration, number of sprays, specification of the soil/substrate type, the effect on infection rate and type of infection are mentioned. The reduction in the infection rate is specified as: + moderate (<30%), ++ good (30–70%) and +++ very good (>70%). The concentration of the KSi in the study on strawberry plants was not specified.

Crop, Monocot (M)/Dicot (D)	Product & Concentration	No. of Sprays	‘Soil’ Type	Effect on Infection Rate	Type of Infection
**Cucumber; D**	KSi: 10, 30 mM	1	rockwool	++	Powdery mildew
**Cucumber; D**	KSI: 10, 20, 30 mM	1 or 2	Container (with soil)	++	Powdery mildew
**Cucumber; D**	KSi: 28, 56 mM	1 or 2	rockwool	+++	Powdery mildew
**Grape; D**	17 mM	1	container	++	Powdery mildew
**Strawberry; D**	KSi conc. not specified	5	field	++	Powdery mildew
**Rice; M**	KSi: 1, 2, 4, 8 and 16 g/L	1	container	++	Rice blast
**Rice; M**	KSi: 40 g/L	1	container	++	Brown spot
**Rice; M**	NaSi: 1, 2 and 3 mM	1	container	++	Rice blast
**Soybean; D**	KSi: 8, 20, 40 and 60 g/L	1	container	++	Soybean rust
**Wheat; M**	KSi: 1.7 mM	6	container	+	Powdery mildew
**Beans; D**	KSi: 8, 20, 40 and 60 g/L	3	field	++	Angular leaf spot
**Capsicum; D**	KSi: 7.5 mM	daily	field	+	Anthracnose
**Chili, D**	KSi: 1, 2, 4 mL/L	3	field	++ ++	Leaf spot & Powdery mildew

**Table 2:** The effects of applying AB Yellow^®^ (PEG-sSA (2.5%) plus boric acid (0.4%), molybdenum (0.1%) and zinc (1.5%), to several crops during the period 2012 to 2017.

Crop	Year	Country	Yield	Type/(No. of Trials)	Remarks
**Chili Peppers**	2012	India	+39%	RCBD (2)	Reduced infections
**Tomato**	2012	India	+31%	RCBD (2)	Brix: ++
**Eggplant**	2012	India	+44%	ET	Greater firmness
**Finger Millet**	2012	India	+39%	RCBD (2)	Reduction of the blast fungus: 58%
**Sweet Corn**	2013	India	+34%	ET/FT (2)	Reduced infections
**Watermelon**	2013	India	+38%	ET/FT (2)	Reduced infections
**Cardamom**	2013	India	+26%	FT	Reduced infections
					Soil pH = 4.8
**Wheat**	2013	Romania	+340%	FT (Figure 1)	Very saline soil
	2014	Ukraine	+19%	FT	Protein content ++
	2015	The Netherlands	+5%	FT	Protein content ++
	2015	Algeria	+37%	FT	Protein content ++
**Rice**	2017	India	+46%	FT (Figure 2a,b)	Very acidic soil

Abbreviations: in column ‘Type of trial’: RCBD (randomized complete block design with at least 3 replications), ET (extension trials in cooperation with scientific institutes) and FT (farmer’s trials in cooperation with scientific institutes). In the column ‘Remarks’ the results show the efficacy against abiotic stress (acidic and saline soil), biotic stress (decreased infection rate) and on quality, e.g., significantly higher brix, TSS, lycopene and vitamin C content (tomato), protein content (wheat) and fruit firmness (eggplant).

**Table 3:** Schematic overview of the effects of the different foliar sprays with (a) silicates; (b) stabilized silicic acid; and (c) nano-silica on 1. Infections (biotic stress), 2. Abiotic stress, 3. Root growth, 4. Plant growth, 5. Leaf size, 6. Yield and 7. Quality of the produce. The symbols used in these columns are specified as: - no effect, + positive effect, ++ good effect and ND = no sufficient data available.

Effects	Silicates	Stabilized Silicic Acid	Nano-Silica
**1. Infections (Biotic stress)**	+/++	+/++	+
**2. Abiotic stress**	-	++	ND
**3. Root growth**	-	+/++	-
**4. Plant growth**	-	+/++	+
**5. Leaf size**	-	++	+
**6. Yield**	-	+/++	+
**7. Quality of the produce**	-	+/++	+
